# Poverty in old age in times of COVID-19—Empirical results from Austria

**DOI:** 10.3389/fpubh.2022.972076

**Published:** 2022-11-17

**Authors:** Lukas Richter, Theresa Heidinger

**Affiliations:** ^1^Department of Social Sciences, St. Pölten University of Applied Sciences, St. Pölten, Austria; ^2^Department of Gerontology and Health Research, Karl Landsteiner University of Health Sciences, Krems an der Donau, Lower Austria, Austria

**Keywords:** poverty in old age, Austria, inequality, COVID-19, AROP, Altersarmut, life situation, vaccination

## Abstract

Early in the pandemic, researchers were cautioning that COVID-19 and the associated health policy countermeasures would have an increased negative impact on groups that were already vulnerable before the pandemic. One of these groups are older people affected by poverty, who according to official figures make up 13.9% of older population in Austria. Even before the pandemic, their living situation was considered precarious. Not without reason, this group has been identified as a high-risk group of the pandemic, due to their increased likelihood of severe COVID-19 related illness and their limited monetary resources and thus lower chances of coping with the pandemic. Nevertheless, research on this group has remained sparse to date. Therefore, the aim of the study is to focus on older people (60+ years) below the poverty line and to compare them with non-poor individuals. Data from the SHARE (Survey of Health Aging and Retirement in Europe) project is used, combining data from the two SHARE Corona Surveys (summer 2020 and summer 2021) and the SHARE Corona Special Austria Survey (December 2020) to gain the most complete picture of life situation during the pandemic. Results demonstrate that older people in poverty were more likely to report poor subjective health before as well as during the pandemic yet were significantly more likely to refuse vaccination against COVID-19, despite adhering to other measures against the pandemic to the same extent as non-poor people. Restrictions in the health care system affected both groups equally and no significant differences in the frequency of social contacts could be found. However, older people below the poverty line were significantly more likely to rely on social support to obtain necessities during the pandemic and were less likely to use the internet. Together, these results point out that disadvantage exist for the older poor in some but not all areas of life during the pandemic. This paper is aimed at providing first insights into the lives of poor older persons during a taxing time and may perhaps inspire more in-depth study of this particularly understudied population.

## Introduction

From a gerontological perspective, the COVID-19 pandemic presents a serious challenge for older people, who are considered a risk group due to a higher probability of severe course of disease and risk of mortality in case of infection (1) which have been related to age-related physiological changes and a higher prevalence of comorbid conditions (2). Additionally, many of the protective measures, taken in part with reference to protecting older people (3), have had far-reaching consequences in other areas of life (4). Systematic reviews and longitudinal studies show a decline in physical activity (5), mental (6) and physical health (7) as well as an increase in social isolation and loneliness (8) due to effects of the pandemic and it's countermeasures. These studies provide vital insights on the lives of older people during the pandemic. It must be pointed out however, that older persons oftentimes are assumed to be a homogeneous group in the scientific as well as in socio-political discourses, despite gerontological admonitions (9, 10). In fact, older people are a most heterogeneous group that differs, among other things, due to different abilities and limitations, biographies, and lifestyles, as well as socio-economic resources and thus their possibilities for action. Taking this heterogeneity into account, it is counterintuitive to assume that older people experience the COVID-19 pandemic in a uniform way or that all are confronted with the same problems and obstacles to an equal extent – for instance Whitehead and Torossian (11) identified different patterns of stressors and joys of the pandemic dependent on socio-economic determinants of older people.

At the beginning of the pandemic, researchers cautioned that the pandemic may have a more profound negative impact on groups that had already been vulnerable prior to the pandemic (12–14), one of which being older people affected by poverty. It is easily overlooked that 16.1% of older people in the European Union and 13.9% in Austria lived below the line of poverty even before the start of the pandemic (15). Despite these early warnings from the scientific community of further precarisation in the pandemic, scientific research on the effect of low income or poverty among older people has remained limited to date, even though as Valtorta and Hanratty (16) ascertained in a literature review, older people from lower socio-economic backgrounds are less financially resilient to shocks such as illness and experience greater financial stress as a result (17, 18). There are few studies explicitly addressing poverty in old age in times of the pandemic, most findings on the situation of older persons living in poverty have to be inferred from gerontological studies which include income or wealth as a control variable. Therefore, the aim of this paper is to inform on the lives of old people living below the poverty line during the pandemic and comparing the situation faced by poor vs. non-poor older (60+) people in Austria.

### Life of older people living in poverty before the pandemic

Prior to touching on the current state of research in the pandemic, we briefly present general findings on poverty in old age. It should be mentioned at the outset that even within Europe the at-risk-of-poverty rate^1^ for older (the age of 65+ is usually assumed) people varies widely (20), as pension systems differ considerably due to specific national designs of multi-pillar pension systems (21). Coupled with social benefits, differences in accessibility (e.g., due to costs) or availability of health care, housing, etc., the life situation of older people living in poverty varies at the national and even local level. Inequalities connected to economic status have been empirically proven, this can be shown by the example of health status: significant correlations have been shown between frailty and material deprivation (22) as well as an increase of multimorbidity with decreasing income (23), which finally culminate in different life expectancies of the lifetime rich as compared to the lifetime poor (24). Furthermore, significantly lower life satisfaction (25, 26) and wellbeing (27) as well as a higher probability of depressive symptoms (28, 29) have also been identified among older people in poverty. Turning to exclusion processes before the pandemic: Barnes et al. (30) stated that older people in the lowest income quintile are more often excluded from financial products, material goods and experience neighborhood exclusion as well as exclusion from social relationships. This accumulation of disadvantage is particularly problematic as social support for example is highly relevant for older people living in poverty as it helps to overcome challenges in everyday life (31) caused, for instance, by health restrictions (18).

To recapitulate, it can be said that older people living in poverty are confronted with disadvantages and precarious life situations, which are either due to or influenced by their economic status. It must be said, that precarity fortunately is not universally found in the poor as many have been able to develop coping strategies. An important explanatory factor for disadvantages is the persistence of poverty among older people – at least in Austria. Even though a certain income dynamic in old age does exist, Jensen and McLaughlin (32) state that income changes often occur on a small scale. The centrality of the state pension in old age as an expression or result of earned income in the employment phase and the structure of the pension system contribute to a largely steady income situation in old age in Austria – unless of course, changes occur to the household composition or marital status. In Austria 151,000 of the 210,000 poor older people in 2019 had been previously classed as such for at minimum 2 years between 2016 and 2018 (33). This persistent monetary precariousness reduces the chances of coping with crises - rather persons are forced to draw on their limited material and immaterial resources. Approximately 30% of older people below the poverty line in Austria report not being able to save small amounts of money (even as low as 15 Euro) and 35% indicate a larger income as a necessary minimum income than they currently have at their disposal (Statistic on Income and Living Conditions - SILC 2019 - own calculations). In consequence unexpected expenses oftentimes cannot readily be covered and sometimes necessitate “disjunctive decision-making” (34). In short, unexpected expenses (e.g., medical needs, if not covered by health insurance) can only be met by cuts (18, 31) in the socio-cultural subsistence level (e.g., foregoing food or heating). The latter manifests in the non-utilization of the health care system despite actual needs (35) or reduced opportunities in care and nursing (36). In consequence, impoverished older people have a significantly lower chance of recovering from illness or disease than non-poor persons (37).

### Life during the pandemic

With these findings in mind, we turn to the effect of economic status on the older persons life situation during the pandemic. As already mentioned, studies that explicitly deal with poverty in old age during the pandemic are sparse with insights being mostly based on indirect findings (i.e., studies investigating poverty and controlling for age or gerontological studies which include income or financial burden as a control variable). Although inequality or poverty research has dealt intensively with the impact of the pandemic in the overall population, for example with regard to the living situation (38–40) or probability of infection and mortality (41, 42) we focus on results from gerontological research, as these are suited to show how lives of older people in poverty and non-poor older people differ over the course of the COVID-19 pandemic.

A study carried out in the U.S. at the beginning of the pandemic, which mainly, although not exclusively, dealt with older people, was able to show that American respondents below the poverty line were significantly more likely to assume that they would not fall ill from COVID-19 (43) with the result remaining stable in a follow-up survey using the same respondents in the bivariate, but not in multivariate analysis (44). These assumptions could be shown to be false in empirical studies: early results from Sweden using microdata show a 1.35-fold higher mortality risk for older people in the lowest income tertile (45) [see also results from Belgium (46) or from Mexico (47)]. As expected, due to a higher likelihood of poor health, the mortality risk was shown to be higher among the older people below the poverty line.

Against this background, the question arises whether poor older individuals were more likely to adhere to protection measures against COVID-19 infection. Delerue-Matos et al. (48) interpreted the reduction of some social activities which was more probable in older people with financial difficulties than those without difficulty as a precautionary behavior; in contrast focussing on hygienic prevention measures Litwin and Levinsky (49) reported a negative association with better financial capacity. The two contrasting results can be explained by the fact that older people in poverty were already less engaged in (social) activities before the pandemic and therefore may have remained less engaged during the pandemic (50). Paradoxically, this inactivity can be seen on the one hand as an advantage in the pandemic, as costly measures [such as face masks as mentioned in Portacolone et al. (51)] may thereby have been used slightly less often. A problematic finding in this context is, that vaccination hesitancy was significantly higher among older adults reporting problems making ends meet or at risk of poverty (52, 53) at least in the first year of the pandemic. On the other hand reduced (social) activities may have also brought about negative effects: older people with difficulties to make ends meet had a significantly higher probability of feeling depressed (54, 55), anxious (55) and lonely since the outbreak of the pandemic (54, 56) and more often reported decreasing mental health (57, 58).

Cross country analysis of Europe additionally shows a significant higher risk of forgoing care for fear of contracting COVID-19 and a higher risk of being unable to obtain a medical appointment in the first months of the pandemic (59–61), although accessibility differs between European countries (62). Twelve percent of older people with difficulties making ends meet postponed regular payment of bills and 27% dipped into their savings (63) – unfortunately the later study didn't compare the results with non-poor older people. It is important in this context, that, although many older people receive a relatively stable pension, they also have had to face income losses from paid employment in addition to changes in household expenditure: results from the Survey of Health, Aging and Retirement show that older people (50+) with low income more frequently reported a job loss (64) or working less hours since the outbreak of the pandemic (65).

In summary, older people with low financial means seem (more) negatively affected by the pandemic than older persons without financial difficulty. However prior results are sparse and often must be extracted from large multivariate studies which do not focus on the topic of poverty explicitly. Complicating this further is the fact that the measurement concept of poverty differs across studies with some using indicators on financial difficulties (such as the ability to make ends meet) and other opting for a categorisation of income within the used sample. This constitutes the main difficulty for not being able to relate many of these study results with the frequently used monetary poverty concept “at risk of poverty” as used in the European Union or Eurostat. This paper therefore deals explicitly with older people below the at-risk-of-poverty threshold and compares them to non-poor older persons in multiple dimensions of life during the pandemic such as health status, adherence to protective measures and perceptions and experiences related to the virus.

## Methods

### Sample

Survey data from three waves of the longitudinal Survey of Health, Aging and Retirement Study (SHARE) were combined in order to achieve the most accurate picture of life during the pandemic: data from the summer 2020 Corona survey 1 (SCSS20) was combined with the winter 2020 Corona survey (special survey of the Austrian study population- SCSAT20) as well as the summer 2021 Corona survey 2 (SCSS21); all the analyzed datasets are based on version 8.0.0 (66, 67). Normally, the survey is conducted *via* a face-to-face Computer Assisted Personal Interview (CAPI), but the pandemic forced a switch to Computer Assisted Telephone Interviews (CATI). Data at the three timepoints were therefore collected *via* CATI (68). Furthermore, sociodemographic information (age, household size) as well as information on household income was supplemented by importing information from wave 8 of the SHARE survey, conducted in 2019 (69, 70), or other most recently completed surveys, in order to achieve maximal explanatory power in the variables of socio-economic status. Persons were excluded from analysis if they did not take part in all three Corona surveys. A detailed coding plan for all analyzed variables can be found in Supplementary Table A.1. Additionally, only persons 60 and above were included. This threshold was chosen as this age constitutes the average retirement age in Austria.

### Variables

The main variable of interest relates to whether a respondent is classed as income poor. To calculate this distinction the most recent information on the economic situation of the individual was used. Household income equivalency was computed for each participant and compared to the EU-SILC 2020 threshold for risk of poverty in Austria (15,933 Euro/year) (71). Participants were then classed as been “non-poor” or “poor.” Validity of this variable is supported by a moderate correlation with the variable “being able to make ends meet” (*Cramer's V* = 0.336, *p* < 0.001). As the different survey waves included varying items, an overview of the variables their original and recoded manifestations as well as their survey wave of origin are presented in Supplementary Table A.1 (see Appendix).

#### Perception and own experience with the virus

Variables discussing the perception of the COVID-19 virus were included solely in the winter 2020 survey (SCSAT20) and covered the estimated probability of catching the virus (“How high do you estimate your risk of catching Corona within the coming 6 months?”) as well as the estimated severity of COVID-19 illness (“How dangerous would a Corona infection be for you considering your health?”). Furthermore, participants were asked to inform on past COVID-19 infections in summer 2020 (SCSS20) and summer 2021 (SCSS21) – using both outcomes two groups were formed: respondents indicating a positive COVID-19 test vs. respondents without a positive COVID-19 test since the outbreak of the pandemic.

#### Vaccination willingness

Willingness to get vaccinated was surveyed in the winter 2020 survey (“If a vaccine against COVID-19 were available, would you get vaccinated?”) as well as in the summer 2021 survey which included questions on realized COVID-19 vaccination (“Have you been vaccinated against COVID-19?”) as well as ambition to get vaccinated (“Would you want to get vaccinated against COVID-19?”). Information of these two variables was combined to form the variable vaccination willingness in summer 2021 which combined persons who already had received their vaccination and those who were planning to get vaccinated to compare against those who were not willing or unsure about getting a vaccination. Attitude change toward vaccination between winter 2020 and summer 2021 was calculated and persons were classed as follows: consistently accepting of a vaccination, consistently rejecting vaccination, consistently unsure about vaccination, switch from rejection to acceptance, switch from unsure to acceptance, and switch from acceptance to rejection or uncertainty between timepoints.

#### Compliance

Variables describing the compliance with the pandemic mitigation measures included wearing a face mask in public, keeping a distance from others in public, washing the hands more frequently than usual and using hand sanitizer or disinfectant fluids more frequently than usual. Compliance with these measures was surveyed in summer 2020, in winter 2020 questions on the reduction of social contact were introduced (“Did you reduce your social contacts with people outside of your household at the beginning of the pandemic as well as at the time of the survey?”). Finally, the use of COVID-19 tests, a service put in place to prevent the spread of the virus, was surveyed retrospectively in the summer 2021 survey (“How many times have you been tested for COVID-19?”).

#### Health status

Information on subjective health status was collected in summer 2020 and summer 2021. In the summer 2020 survey, subjects were asked to compare current health to the time before the outbreak of the pandemic; current health status was collected in the summer 2021 survey. Mental health was assessed in the winter 2020 survey. This included the Euro-D Scale (72) which informs on feelings of depression in late-life (range 0 “not depressed” to 12 “very depressed”) as well as the GAD-7 scale by Spitzer et al. (73) which is a well-established, brief measure for assessing generalized anxiety disorder (range 0 “no anxiety” to 21 “high anxiety”). Additionally, the use of health care services was included to capture health behavior in the pandemic. Questions on forgoing or postponing medical appointments or being denied medical appointments were asked in the summer 2020 (retrospectively spanning the in time since the outbreak) and summer 2021 (retrospectively spanning the time since summer 2020) surveys. Furthermore, visits to hospital as well as to medical practices and other medical facilities were queried in summer 2021.

#### Social participation

Social participation during the pandemic was also analyzed for differences between poor and non-poor persons. Contact frequency to children, grandchildren and neighbors/friends/colleagues was surveyed in summer 2021. Information on face to face but also electronic contact was collected. Social support was also queried, whereby persons were asked to report whether they had received help in obtaining necessities by children, other relatives, or friends/neighbors/colleagues.

#### ICT use

Finally, the use of information and communications technology (ICT) was examined according to economic status in old age. Persons were asked whether they had (a) used the internet since the outbreak of the pandemic. If they answered affirmatively, they were asked whether they had used the internet in order to (b) find information on health-related issues, (c) gain information about government services (d) manage their finances and (e) buy or sell goods/ services. Furthermore, the use of remote medical services during the pandemic was queried. All information on ICT use was collected in the summer 2021 survey, participants were asked to retrospectively report ICT use in the time since the outbreak.

### Analyses

The analysis was carried out using IBM SPSS 27. Bivariate comparisons of persons classed as ‘poor' compared to those who were not classed as such on discrete variables were done by using Chi^2^ tests (cross-sectional design). *Post-hoc* group comparisons were done using *z*-test with Bonferroni correction. Group comparisons on continuous variables were done *via* unpaired *t*-tests or if necessary, the non-parametric Mann–Whitney *U*-Test. All statistical testing was done using the significance level of α = 0.05. Effect sizes were provided for all computations. A conscious decision was made not to perform multivariate procedures as the data was collected using differing questions and introducing or omitting specific questions at the different timepoints (an overview can be found in Supplementary Table A.1 of the Appendix). Because of this, no statements on the correlation of specific dependent variables and poverty can be made, instead this paper provides comparisons between poor vs. non-poor older individuals at three separate timepoints during the pandemic and thus is primarily exploratory or descriptive in nature.

## Results

### Sample composition

The final sample was comprised of 2,078 persons, due to missing values the sample for analysis was reduced to 1,862 persons, whereof 18.1% were classed as income poor and ~10% reported at least some difficulty in making ends meet. A more detailed description of the sample structure can be found in the adjoining Table 1.

**Table 1:** Sample structure.

	**Distribution in sample**
**Gender**	
Male	39.0%
Female	61.0%
**Age**	
Metric	*Mean* = 73.31 years, *SD* = 7.97 years
**ISCED 97**	
Classification from 0 (no formal education) – 6 (high formal education)	*Mean* = 3.33, *SD* = 1.30
**Household size**	
Metric	*Mean* = 1.8 persons, *SD* = 0.8 persons
**Income poor**	
Yes	18.1%
No	81.9%
**Make ends meet**	
With great difficulty	1.4%
With some difficulty	8.8%
Fairly easily	36.3%
Easily	53.4%

Gender is restricted to two categories in the SHARE surveys.

ISCED, International Standard Classification of Education.

#### Perception and experience with the coronavirus

Poorer participants indicated a significant but marginally higher risk of becoming infected with the Coronavirus than those in the non-poor group even though most of both groups estimated to be at (very) low risk of infection: 68.5% non-poor vs. 61.9% poor participants (Table 2).

**Table 2:** Perception of and experience with the coronavirus.

**Risk of catching corona (SCSAT20)**			**Dangerous for own health (SCSAT20)**		
	**Non-poor**	**Poor**		**Non-poor**	**Poor**
*(very) Low risk*	68.5%_a_	61.9%_b_	*Not/a bit dangerous*	15.4%_a_	16.5%_a_
*Medium risk*	26.5%_a_	30.6%_a_	*Medium dangerous*	31.1%_a_	24.8%_b_
*(very) High risk*	5.0%_a_	7.5%_a_	*Quite/very dangerous*	53.5%_a_	58.7%_a_
*n*	1,448	314	*n*	1,413	315
*Cramer's V*	0.060		*Cramer's V*	0.054	
*p*	0.048		*p*	0.082	
**COVID-19 infection in the past until summer 2021 (SCSS20** + **SCSS21)**					
	**Non-poor**	**Poor**
*No*	95.1%_a_	95.3%_a_
*Yes*	4.9%_a_	4.7%_a_
*n*	1,525	337
*Cramer's V*	0.054	
*p*	0.082	

The lower-case letters in the tables show the result of the z-test. aa = no significant difference between poor and non-poor; ab = significant difference between categories. We recommend interpreting the z-test only if the respective chi^2^ test in the table is significant (when *p* < 0.05). *Cramer's V* measures the strength of the relationship between variables, *n* = sample size, SCSS20 = SHARE Corona Survey – summer 2020, SCSS21 = SHARE Corona Survey – summer 2021. SCSAT20 = SHARE Corona Special Austria Survey – winter 2020.

Neither the estimation of danger nor the comparison of past infection showed a statistically significant difference which can be interpreted as there being no disadvantage of low socioeconomical status on experience with the virus. For both groups the majority of participants considered COVID-19 to be a potentially serious threat to their health (53.5% non-poor, 58.7% poor participants). The high number may not be surprising here, as the survey took place mainly in December of 2020, shortly after the second wave of infection had reached its peak in Austria when the number of hospital admissions and deaths per day were at an all-time high (74, 75). However, seen from the current perspective, the 7-day incidence remained relatively low until the summer of 2021, with the highest number of newly identified cases of confirmed SARS-CoV2 infection being ~560 per 100,000 inhabits (on 12.11.2020). For this reason, the low number of positive tests (aka evidenced infections) in the sample (5% of all respondents with no significant differences between groups) can be explained.

#### Vaccination willingness

The free vaccination against COVID-19 had been promoted relatively early on in Austria with the first persons receiving a vaccination as early as December 27 2020, with abundant media attention (76). However, willingness to get vaccinated in winter of 2020 remained ambiguous with 55.1% of all participants indicating that they would like to receive a vaccination while 22.6% declined wanting to get vaccinated and the remaining 22.4% indicated feeling unsure about a vaccination (Table 3).

**Table 3:** Vaccination willingness.

**Vaccination willingness winter 2020 (SCSAT20)**				**Vaccination willingness summer 2021 (SCSS21)**			
	**Non-poor**	**Poor**	**Total**		**Non-poor**	**Poor**	**Total**
*Vaccinated, ready to be vaccinated*	58.9%_a_	37.7%_b_	55.1%	*Vaccinated, ready to be vaccinated*	91.5%_a_	80.8%_b_	89.6%
*Refusal*	19.9%_a_	34.7%_b_	22.6%	*Refusal*	4.9%_a_	14.7%_b_	6.6%
*Unsure*	21.2%_a_	27.6%_b_	22.4%	*Unsure*	3.6%_a_	4.5%_a_	3.8%
*n*	1,524	337	1,861	*n*	1,522	334	1,856
*Cramer's V*	0.171			*Cramer's V*	0.154		
*p*	< 0.001			*p*	< 0.001		

The lower-case letters in the tables show the result of the z-test. aa = no significant difference between poor and non-poor; ab = significant difference between categories. We recommend interpreting the z-test only if the respective chi^2^ test in the table is significant (when *p* < 0.05). *Cramer's V* measures the strength of the relationship between variables, *n* = sample size, SCSAT20 = SHARE Corona Special Austria Survey- winter 2020, SCSS21 = SHARE Corona Survey – summer 2021.

Comparing poor vs. non-poor participants showed a stark difference with persons classed as poor indicating far more unwillingness (34.7 vs. 19.9% in non-poor persons) or uncertainty (27.6 vs. 21.2% in non-poor persons) to get vaccinated against the virus (*Cramer's V* = 0.17, *p* < 0.001) in the winter of 2020. By the summer of 2021 most persons were already vaccinated with 6.6% of all participants continuing to decline a vaccination and another 3.8% stating that they were unsure whether they would like to receive a vaccination in the future. Comparing poor vs. non-poor persons showed significant differences between the groups (*Cramer's V* = 0.154, *p* < 0.001): poor persons were significantly more likely to be vaccination rejectors (14.7 vs. 4.9% of non-poor group), for the group of undecided persons, no difference across groups could be found.

**Figure 1:**
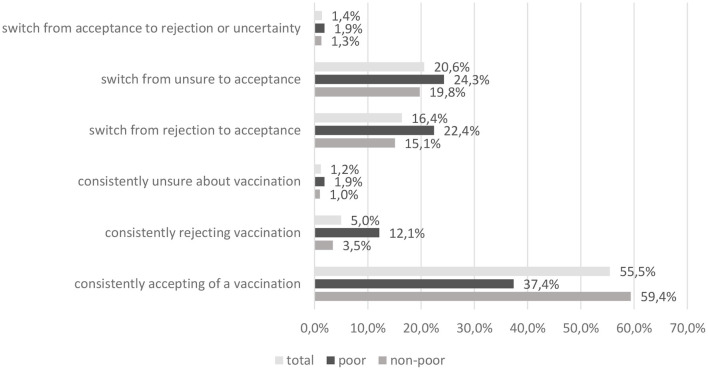
Attitude change toward vaccination.

Attitude change toward vaccinations was analyzed for the entire sample. Five percent of rejectors in winter 2020 remained rejectors in summer 2021, 1.2% of the previously uncertain remained in summer 2021 (Figure 1). Most change was seen from uncertainty in 2020 to acceptance in 2021 (20.6%) additionally 16.4% of rejectors in 2020 indicated an accepting stance toward the vaccine in 2021. Poor and non- poor persons differed significantly in attitude change (*Cramer's V* = 0.26, *p* < 0.001). Of the group of poor participants significantly more persons remained firm rejectors than from the non-poor (12.1 vs. 3.5%), however more rejectors also switched to acceptance from this group (22.4 vs. 15.1%). This fact is unsurprising seeing as the group of vaccination rejectors was far larger in the group of poor participants as compared to the non-poor in winter 2020. These variables show a differential picture of vaccination acceptance between the financially better off vs. poorer persons. Additionally, when subjective health was included (not shown here), 15% of old poor persons who indicated fair/poor health refused a COVID-19 vaccination in summer 2021 (*Cramer's V* = 0.172 *p* < 0.001).

#### Compliance

Most of the sample indicated being compliant with the pandemic mitigation measures. Comparing the groups of poor vs. non-poor participants showed no significant differences in compliance with all queried measures except for “using hand sanitizer more frequently” which was indicated less in the poor group (77% agree vs. 84.3% agree in non-poor group, *Cramer's V* = 0.075, *p* < 0.001). Comparing compliance to the measure “reduction of social contacts” (surveyed in summer 2020) yielded no significant difference between groups (Table 4), comparing the use of the COVID-19 tests however showed group differences: older people in poverty were twice as likely to have never used a COVID-19 test than those classed as non-poor (12 vs. 6.6%). Another difference could be seen in the “most frequent testers” (10 or more test) where non-poor were significantly more likely (29.5 vs. 12.3%) to have used a higher number of tests (*Cramer's V* = 0.17, *p* < 0.01).

**Table 4:** Compliance.

**Summer 2020 (SCSS20)**	**Wore a face mask in public**			**Kept distance from others in public**		
		**Non-poor**	**Poor**		**Non-poor**	**Poor**
	*Always*	72.0%_a_	74.7%_a_	*Always*	80.5%_a_	80.1%_a_
	*Often*	22.7%_a_	18.2%_a_	*Often*	16.8%_a_	15.2%_a_
	*Sometimes*	4.8%_a_	5.7%_a_	*Sometimes*	2.2%_a_	4.4%_b_
	*Never*	0.4%_a_	1.4%_a_	*Never*	0.5%_a_	0.3%_a_
	*n*	1,433	296	*n*	1,432	296
	*Cramer's V*	0.062		*Cramer's V*	0.053	
	*p*	0.085		*p*	0.181	
	**Washed hands more than usual**			**Hand sanitizer or disinfection more than usual**		
		**Non-poor**	**Poor**		**Non-poor**	**Poor**
	*Yes*	89.4%_a_	86.1%_a_	*yes*	84.3%_a_	77.0%_b_
	*No*	10.6%_a_	13.9%_a_	*no*	15.7%_a_	23.0%_b_
	*n*	1,525	337	*n*	1,525	335
	*Cramer's V*	0.041		*Cramer's V*	0.075	
	*p*	0.074		*p*	0.001	
**Winter 2020 (SCSAT20)**		**Currently reduce your social contacts**							
				**Non-poor**	**Poor**
			*Yes*	94.9%_a_	92.6%_a_
			*No*	5.1%_a_	7.4%_a_
			*n*	1,521	337
			*Cramer's V*	0.040	
			*p*	0.086	
**Summer 2021 (SCSS21)**	**Number of times tested for COVID-19**							
				**Non-poor**	**Poor**
			*Not at all*	6.6%_a_	12.0%_b_
			*Only once*	7.6%_a_	13.5%_b_
			*2–5 times*	36.2%_a_	38.7%_a_
			*6–10 times*	20.0%_a_	23.4%_a_
			*More than 10 times*	29.5%_a_	12.3%_b_
			*n*	1,522	333
			*Cramer's V*	0.17	
			*p*	< 0.001	

The lower-case letters in the tables show the result of the z-test. aa = no significant difference between poor and non-poor; ab = significant difference between categories. We recommend interpreting the z-test only if the respective chi^2^ test in the table is significant (when *p* < 0.05). *Cramer's V* measures the strength of the relationship between variables, *n* = sample size, SCSS20 = SHARE Corona Survey – summer 2020, SCSAT20 = SHARE Corona Special Austria Survey- winter 2020, SCSS21 = SHARE Corona Survey – summer 2021.

#### Reported health

Most participants reported excellent to good health prior to the pandemic, one quarter reported having fair to poor health preceding the outbreak of COVID-19 (Table 5). Current health status was rated as “good” by 38% of participants, 32% assessed their health as being “fair/ poor” in the summer of 2021. Therefore, health seemed to have declined in a number of participants, which may be explained by the effects of the crisis however, due to the extended length of the pandemic, could also show a natural decline in health with increasing age. Comparing the two groups showed a disadvantage for poor older people before and during the pandemic. Persons classed in this category were significantly less likely to assess their current health as “excellent/very good” (21 vs. 35%) and were more likely to report “fair/poor” health (36%−23% in non-poor group) prior to the outbreak (*Cramer's V* = 0.141, *p* < 0.001). They were also more likely to report fair/poor health in the pandemic (summer 2021) with 41% as compared to 30% (*Cramer's V* = 0.010, *p* < 0.001).

**Table 5:** Reported health.

**Subjective health before the outbreak (SCSS20)**				**Subjective health in summer 2021 (SCSS21)**			
	**Non-poor**	**Poor**	**Total**		**Non-poor**	**Poor**	**Total**
*Excellent/very good*	35.1%_a_	20.8%_b_	32.5%	*Excellent/very good*	31.9%_a_	22.0%_b_	30.1%
*Good*	42.2%_a_	43.3%_a_	42.4%	*Good*	38.4%_a_	37.1%_a_	38.1%
*Fair/poor*	22.7%_a_	35.9%_b_	25.1%	*Fair/poor*	29.8%_a_	40.9%_b_	31.8%
*n*	1,525	337	1,862	*n*	1,525	337	1,862
*Cramer's V*		0.141		*Cramer's V*		0.104	
*p*		< 0.001		*p*		< 0.001	

The lower-case letters in the tables show the result of the z-test. aa = no significant difference between poor and non-poor; ab = significant difference between categories. We recommend interpreting the z-test only if the respective chi^2^ test in the table is significant (when *p* < 0.05). *Cramer's V* measures the strength of the relationship between variables, *n* =sample size, SCSS20 = SHARE Corona Survey – summer 2020, SCSS21 = SHARE Corona Survey – summer 2021.

Mental health was approximated with information on depression (Euro-D) (76) and anxiety (GAD-7) (77). Using the cut-off for the Euro-D scale used in the majority of SHARE studies (<4 “not depressed,” 4–12 “case of depression”), 72% of all participants were classified as “not depressed” during the pandemic. Comparison of the two groups showed a significantly higher mean among the income poor (2.06) vs. the non-poor group (1.80). Since statistical requirements for parametric testing were not fulfilled, a Mann–Whitney-*U-*test was carried out to test statistical significance. This showed a small but significant difference between poor vs. non-poor persons: *U* = 223,434.000, *p* < 0.001, *r*= 0.089. In addition, most participants report few symptoms of anxiety: Using the GAD-7 scoring system 85% of the sample were classed as having “no or minimal anxiety,” 13% could be classed as having “slight anxiety.” Comparing both groups, a small effect (*Cramer's V* = 0.086, *p* < 0.001) can be stated: significant more older people above the poverty line reported “no or minimal anxiety” (86 vs. 78%).

#### Health care utilization

Examining limitations to the health care services during the pandemic showed that older people could partly not make use of health services (data taken from the summer 2020 survey): 14% of all participants reported forgoing, 27% postponing treatment due to COVID-19 and 5% reported having been denied an appointment. In the 2021 summer survey, the number of participants reporting health care difficulties was lower: 8% forwent an appointment, 11% postponed an appointment and 3% were denied an appointment in the second year of the pandemic. In addition, 28% of the sample were treated in hospital over the course of the pandemic, 82% confirmed having gone to a doctors practice or another medical facility outside of a hospital. Since there were no significant differences between poor/non-poor persons in any of the tested variables the corresponding Supplementary Table A.2 has been moved to the Appendix. In short, limitations in health care use were independent of economic status in Austria among the older population.

#### Social contact and support

Information on social contacts is summarized in the Appendix as well (Supplementary Table A.3), as no significant differences between the groups were found in this block of variables. Most participants reported having had face to face contact with children (69%), grandchildren (53%) or friends/neighbors/colleagues (59%) at least once a week in summer 2021. Electronic contact was found to be even higher with 87% of all participants with children reportedly having electronic contact with them at least once a week. Friends/neighbors/ colleagues were the second most frequent contact group – 75% reported a contact frequency of once a week or higher with this group. Social contact (face to face and electronically) did not differ significantly between poor and non-poor older persons in Austria.

Focussing on social support, 42% of the sample reported having been helped by their children, 15% reportedly leaned on friends/neighbors of colleagues for help obtaining necessities in the pandemic, 8% were helped by other relatives. Differences between poor and non-poor could be seen in the data (Table 6), whereby older people below the poverty line were more likely to receive support from children (49.4 vs. 39.4% non-poor, *Cramer's V* = 0.078, *p* < 0.05) and other relatives (14.9 vs. 7.7% non-poor, *Cramer's V* = 0.10, *p* < 0.001), social support by friends/neighbors/ colleagues did not differ according to economic status.

**Table 6:** Social support.

**Help received from own children (SCSSS21)**			**Help received from other relatives (SCSSS21)**		
	**Non-poor**	**Poor**		**Non-poor**	**Poor**
*Yes*	39.4%_a_	49.4%_b_	*Yes*	7.7%_a_	14.9%_b_
*No*	60.6%_a_	50.6%_b_	*No*	92.3%_a_	85.1%_b_
*n*	1,399	310	*n*	1,453	322
*Cramer's V*	0.078		*Cramer's V*	0.097	
*p*	0.001		*p*	< 0.001	
**Help received from neighbors/friends/colleagues (SCSSS21)**		
	**Non-poor**	**Poor**
*Yes*	14.2%_a_	16.8%_a_
*No*	85.8%_a_	83.2%_a_
*n*	1,494	333
*Cramer's V*	0.029	
*p*	0.221	

The lower-case letters in the tables show the result of the z-test. aa = no significant difference between poor and non-poor; ab = significant difference between categories. We recommend interpreting the z-test only if the respective chi^2^ test in the table is significant (when *p* < 0.05). *Cramer's V* measures the strength of the relationship between variables, *n* = sample size, SCSS21 = SHARE Corona Survey – summer 2021.

#### ICT use

Fifty-six percent of all participants reported using the internet, however, with significant differences between the two groups as shown in Table 7 (*Cramer's V* = 0.175, *p* < 0.001). Only 37.2% of older people in poverty use the Internet, indicating a significant digital gap which persisted during the pandemic.

**Table 7:** ICT use.

	**Usage of internet since the outbreak (SCSS21)**	
	**Non-poor**	**Poor**
*Yes*	59.8%_a_	37.2%_b_
*No*	40.2%_a_	62.8%_b_
*n*	1,525	336
*Cramer's V*	0.175	
*p*	< 0.001	
**Usage of internet in order to find information on health-related issues (SCSS21)**			**Usage of internet in order to gain information about government services (SCSS21)**		
	**Non-poor**	**Poor**		**Non-poor**	**Poor**
*Yes*	69.3%_a_	72.0%_a_	*Yes*	39.1%_a_	31.5%_a_
*No*	30.7%_a_	28.0%_a_	*No*	60.9%_a_	68.5%_a_
*n*	912	125	*n*	908	124
*Cramer's V*	0.019		*Cramer's V*	0.051	
*p*	0.538		*p*	0.1	
**Usage of internet in order to manage finances (SCSS21)**			**Usage of internet in order to buy/sell goods/services (SCSS21)**		
	**Non-poor**	**Poor**		**Non-poor**	**Poor**
*Yes*	58.6%_a_	49.2%_b_	*Yes*	53.7%_a_	41.9%_b_
*No*	41.4%_a_	50.8%_b_	*No*	46.3%_a_	58.1%_b_
*n*	912	124	*n*	912	124
*Cramer's V*	0.061		*Cramer's V*	0.077	
*p*	0.048		*p*	0.014	
		**Remote medical consultation (SCSS21)**	
		**Non-poor**	**Poor**
*Yes*		7.9%_a_	6.3%_a_
*No*		92.1%_a_	93.8%_a_
*n*		1,525	336
*Cramer's V*		0.024	
*p*		0.31	

The lower-case letters in the tables show the result of the z-test. aa = no significant difference between poor and non-poor; ab = significant difference between categories. We recommend interpreting the z-test only if the respective chi^2^ test in the table is significant (when *p* < 0.05). *Cramer's V* measures the strength of the relationship between variables, *n* = sample size, SCSS21 = SHARE Corona Survey – summer 2021.

It should be noted that the next results refer only to people who reported using the internet in both groups (see *n* in Table 7). Findings revealed that poor and non-poor old internet users differed significantly in use of the internet particularly for the purpose of “managing finances”: 58.6% of non-poor users acknowledge using the internet for this purpose while only 49.2% of all income poor users do (*Cramer's V* = 0.061, *p* = 0.048). Similarly, the latter group were less likely to acknowledge using the internet “to buy/sell goods or services” (41.9 vs. 53.7%, *Cramer's V* = 0.077, *p* = 0.014). Although no significant differences were found, it is interesting to note that only about 30% of users report using the Internet for health-related issues, which, in light of the pandemic, seems quite low. In addition, all older respondents (see *n* in Table 7) were asked whether they used telemedical services during the pandemic. Telemedical care was used sparsely in the sample - 8% of all participants stated that they had used remote medical services at least once during the pandemic. A comparison between both groups yielded no significant result.

## Discussion

The results show that there are no significant or marginal differences in perception of and experience with the coronavirus between older people below and above the poverty threshold. A possible explanation could be that the topic of COVID-19 was strongly represented in the Austrian media with older people, especially at the beginning, being generally addressed as a risk group. This is likely to have influenced the perceptions of the respondents independent of economic status. Looking at experience with the virus, it is noteworthy that positive testing (evidenced COVID-19 infections) was found not to differ between the two groups indicating similar familiarity with the virus. This result however, does not inform on possible differences in mortality or severity of disease, which has been shown to differ between the poor and non-poor in other studies (45–47). Additionally, it must be kept in mind that older people living in poverty reported having undergone significantly less COVID-19 testing up until summer 2021. It is therefore quite possible that some respondents had experienced an undetected infection (without or with mild symptoms). All in all, the result of the different test frequencies provides food for thought: even though Austria has established a generous (and largely free) testing programme, persons living in poverty seem to have been less attainable and or persuadable for this effort. This may be an artifact carried over from the early days of the pandemic when testing was more difficult to access and (often) costly (75, 77). However more research is needed to determine whether the differences are due to continuing barriers to access for older people in poverty. Results regarding compliance show that most of older population strictly adhered to the mitigation measures set forth to decrease viral spread with no differences between older persons of higher or lower social status. With respect to the use of hand sanitizer or disinfectant minor significant differences could be seen with poor persons reporting lower adherence to this mitigation measure. A probable explanation for this difference could be the disparate financial means of the groups: persons living below the poverty threshold may not be able to afford sanitizer or disinfectant products.

Examining health in the pandemic, we see that older people in poverty show a less favorable state of health (55, 56). This is evident in the pre-existing differences on subjective health which also extend into the pandemic. Although the effect size decreased from 0.141 to 0.104, the difference in share of persons classed in the lowest category (fair/poor health) when comparing poor to non-poor persons remained largely unchanged (before outbreak 13.2% points difference vs. 11.1% points difference in summer 2021). A deterioration of subjective health has nevertheless been apparent in the pandemic (78). Although age effects are likely to play a role here, pandemic effects cannot be ignored, which have been shown in previous studies (79–81). Considering mental health indicators, only minor (albeit significant) results emerge with poorer persons exhibiting higher likelihood to report symptoms of depression. No significant differences were found between groups in the limitations to health services of older people in Austria. In other words, cancellations and refusals of appointments were independent of the older person's financial background. This sets Austria apart from other countries in Europe, where the use of the health care system was shown to be more dependent on socioeconomic inequalities (60, 62). This is probably due to the fact that the health care system in Austria remains relatively egalitarian: according to official figures, 99.9% of the Austrian resident population is covered by health insurance (82).

Another positive aspect to note is that older people in Austria were able to stay in touch with their children, grandchildren or friends and neighbors during the pandemic, regardless of income poverty. This may be somewhat surprising in the case of electronic contact, when considering the ongoing costs of use. However, compared to many other European countries, the cost of mobile telephony in Austria is relatively low and usually comes with minute credit^2^ which may have helped poorer individuals stay in touch with their social network. However, older people below the at-risk-of-poverty threshold were significantly more likely to depend on social support to obtain necessities since outbreak, this finding is consistent with previous findings on low income populations (31, 83). Older people reported primarily relying on their children during the pandemic, this is consistent with findings of as studies conducted prior to the pandemic. In addition, the aged poor were significantly more likely to be helped by other relatives, whereas this was not the case with friends. Interestingly, the support of friends plays a considerable role at 15%. All in all, it can be said that the pandemic with its mitigation measures meant that a not unremarkable proportion of older people were dependent on external support. The question must be asked whether the lost autonomy can be regained, especially since a definite end to the pandemic is not foreseeable at this time. An improvement was achieved with the roll out of the vaccination however, which lead to a significant reduction in severe courses of illness and hospital admissions. Most importantly, these results show how important social support is for older people below the poverty line (31, 34), as low financial means limit alternative actions (be it ordering goods or using a car when public transport appears unsafe due to the pandemic). Further analyses are necessary to examine the ways older persons without social support coped with the challenges posed by the pandemic.

The results regarding ICT use continue the pre-pandemic trends (84) showing that internet use among older and poor people significantly lags behind the non-poor older persons also in the time of the pandemic. Although the pandemic must be seen as a strong driver of change, limited financial resources are likely to continue preventing increased ICT utilization (84, 85). Another possibility is that older people living in poverty have not yet recognized the benefits of ICT use, although it must be pointed out that financial resources also counteract simple trial and error. This is underlined by the finding that the few poor respondents who report using the internet during the pandemic use it in much the same way as the non-poor, except for managing finances and online shopping, which seems logical. In summary, the results should draw attention to the importance of continuing to study ICT use among older people with low income or below the poverty line as a lag in these groups continues to exist. We must therefore ask how ICT can be brought closer to these vulnerable groups as the risk of digital exclusion is not only a possibility but a reality in many of their members.

## Conclusion

Overall, the analysis of the life situation of older people below the poverty threshold in Austria presents both light and shadows. In some areas, older people in poverty were able to keep pace with non-poor people during the pandemic, such as in the upkeep of social contact and access to the health care system. It should also be emphasized that older people were very compliant with the majority of mitigation measures. However, findings on vaccination willingness paint a concerning picture with older people below the poverty line being more likely to refuse vaccination despite, as seen in some cases higher health risks due to poorer general health (86). Although many older people had chosen to become vaccinated by summer 2021, continuing deficits were noted among the poor group. As a recent study shows, differences in Austria along financial resources persist even after controlling for education and other factors (53). A mix of factors is probably responsible for this: although vaccinations are free of charge in Austria, they are and have been accessible to varying degrees (e.g., distance to the nearest vaccination center, etc.). From an economic perspective, these varying accessibilities are also associated with varying costs (e.g., travel costs) and may have disadvantaged older people in poverty. In addition, willingness to vaccinate is influenced, for example, by trust in government or proneness to conspiracy theories. Further work is needed to examine how poorer people (and thus often groups with lower education) can be more appropriately addressed and motivated for health measures. Furthermore, central differences between poor and non-poor older persons were evident in the need for social support and ICT use. In both areas, the limited financial resources - which on the one hand necessitate support and on the other hand limit ICT use - are relevant factors.

Finally, some limitations of the study must be acknowledged, the most prominent being that the description of the life situation of the older income poor during the pandemic only included particular variables and therefore cannot be seen as a thorough description of said life situation. Variables were selected according to previous scientific findings as well as data availability. Furthermore, information used for analyses were collected in three sperate surveys (aka three timepoints), limiting generalizability across the span of the pandemic. As the surveys included different variables at different timepoints, no longitudinal analyses could be calculated. Whenever possible (inclusion of the same variable at two timepoints into the survey), change coefficients were calculated to inform on temporal differences (see vaccination willingness). Additionally, this study forwent multivariate analyses overall opting to describe the life situation of the sampled persons as well as comparing poor vs. non-poor individuals in a rich country such as Austria. Against this background, it must also be pointed out that a causal direction between the tested variables and the group membership (poor/non-poor) cannot be assumed apart from logical and theoretical considerations. For example, poor health may have led to poverty and poverty may have led to poor health - studies point to both phenomena or an interaction. For the present study, however, the relevant result is whether there are differences between the groups.

The aim of this study was to give first insights into a sparsely studied field in order to incite interest and possibly initiate further research into better understanding the living situation of a group that is, at least partially, considered vulnerable, during the pandemic and beyond. Study results showed, that while vulnerability of income poor older persons can be seen in a certain share, particularly in some areas, not every poor person was affected by precarisation during the pandemic with many people having learned to cope with limited resources and overcoming crises. However, this should not distract us from continuing to address the issue of old age poverty and to intervene in a socio-politically supportive manner.

## Data availability statement

Publicly available datasets were analyzed in this study. This paper uses data from SHARE Waves 8 and 9 (https://doi.org/10.6103/share.w8.800, https://doi.org/10.6103/share.w8ca.800, and https://doi.org/10.6103/share.w9ca.800), see Börsch-Supan et al. (70) for methodological details.

## Author contributions

LR was the primary author of this manuscript. Analysis and writing were done in collaboration with TH. All authors contributed to the article and approved the submitted version.

## Funding

Under the terms of the Austria Open Access Publishing Framework Agreement, the St. Pölten University of Applied Sciences (Fachhochschule St. Pölten/FH St. Pölten) will cover Article Publishing Fees for eligible authors in any of the Frontiers journals. The SHARE data collection has been funded by the European Commission, DG RTD through FP5 (QLK6-CT-2001-00360), FP6 (SHARE-I3: RII-CT-2006-062193, COMPARE: CIT5-CT-2005-028857, and SHARELIFE: CIT4-CT-2006-028812), FP7 (SHARE-PREP: GA No. 211909, SHARE-LEAP: GA No. 227822, SHARE M4: GA No. 261982, and DASISH: GA No. 283646), and Horizon 2020 (SHARE-DEV3: GA No. 676536, SHARE-COHESION: GA No. 870628, SERISS: GA No. 654221, and SSHOC: GA No. 823782) and by the DG Employment, Social Affairs and Inclusion through VS 2015/0195, VS 2016/0135, VS 2018/0285, VS 2019/0332, and VS 2020/0313. Additional funding from the German Ministry of Education and Research, the Max Planck Society for the Advancement of Science, the United States National Institute on Aging (U01_AG09740-13S2, P01_AG005842, P01_AG08291, P30_AG12815, R21_AG025169, Y1-AG-4553-01, IAG_BSR06-11, OGHA_04–064, HHSN271201300071C, and RAG052527A) and from various national funding sources is gratefully acknowledged (see www.share-project.org).

## Conflict of interest

The authors declare that the research was conducted in the absence of any commercial or financial relationships that could be construed as a potential conflict of interest.

## Publisher's note

All claims expressed in this article are solely those of the authors and do not necessarily represent those of their affiliated organizations, or those of the publisher, the editors and the reviewers. Any product that may be evaluated in this article, or claim that may be made by its manufacturer, is not guaranteed or endorsed by the publisher.

## Footnotes

^1^Eurostat defines the “at-risk-of-poverty rate” as the share of people with an equivalised disposable income below the “at-risk-of-poverty threshold,” which is set at 60% of the national median equivalised disposable income after social transfers (19).

^2^Contracts with 1,000 free minutes and several GB of data volume per month are available for 10 euros or even less in Austria.
