# Supplementary material for: Poverty in old age in times of COVID-19—Empirical results from Austria
Source: Front Public Health. 2022 Nov 17;10:972076. doi: 10.3389/fpubh.2022.972076 (PMC9713516; doi:10.3389/fpubh.2022.972076)
Supplement: Supplementary file 1 [file Data_Sheet_1.docx]

**Appendix**

**Table A.1**

| **Variable block** | **Question (Q) / Generated Variable (GV)** | **Manifestation in Survey** | **Recoded Manifestation in Analysis** | **Survey of origin** |
| --- | --- | --- | --- | --- |
| **perception and own experience with the virus** | Q: How high do you consider your risk of catching Corona within the next 6 months? | 1 (very low) - 5 (very high) | 1-2 = 1 = (very) low risk  3 = 2= medium risk  4-5 = 3 = (very) high risk | SCSAT20 |
|  | Q: How dangerous do you think would a Corona infection be for you considering your health? | 1 (not dangerous at all) - 5 (very dangerous) | 1-2 = 1 = not/ a bit dangerous  3 = 2 = medium dangerous  4-5 = 3 = quite / very dangerous | SCSAT20 |
|  | GV: Covid-19 infection in the past until up summer 2021  GV created by combing data from: SCSS20: variable name CAC005 and SCSS21: variable name CAC105 | 1 = tested positive for Covid-19  5 = tested positive for Covid-19 | 1 = 1 = yes  5 = 2 = no | calculated by combining information from SCSS20 and SCSS21 |
| **vaccination willingness** | Q: If a vaccine against Covid-19 were available, would you get vaccinated? | 1 = yes  2 = no  -1 = don’t know | 1 = 1 = vaccinated, ready to be vaccinated  2 = 2 = refusal  -1 = 3 = unsure | SCSAT20 |
|  | GV: Vaccination readiness in summer 2021  GV created by combining data from: SCSS21: variable name CAHC117 and SCSS21: variable name CAHC118 | 0 = vaccinated  1 = already have a vaccination scheduled  2 = want to get vaccinated  3 = do not want to get vaccinated  4 = still undecided | 0, 1 or 2 = 1 = vaccinated, ready to be vaccinated  3 = 2 = refusal  4 = 3 = unsure | SCSS21 |
|  | GV: Attitude change towards vaccination  GV created by combining the two previous variables (see two lines above) | see two previous lines | 1 = consistently accepting of a vaccination  2 = consistently rejecting vaccination  3 = consistently unsure about vaccination  4 = switch from rejection to acceptance  5 = switch from unsure to acceptance  6 = switch from acceptance to rejection or uncertainty | calculated comparing data from SCSAT20 and SCSS21 |

**Continuation of table A.1**

| **Variable block** | **Question (Q) / Generated Variable (GV)** | **Manifestation in Survey** | **Recoded Manifestation in Analysis** | **Survey of origin** |
| --- | --- | --- | --- | --- |
| **compliance with the pandemic mitigation measures** | Q: How often did you wear a face mask when you went outside your home to a public space? | 1 = always  2 = often  3 = sometimes  4 = never | 1 = 1 = always  2 = 2 = often  3 = 3 = sometimes  4 = 4 = never | SCSS20 |
|  | Q: How often did you keep distance to others when you went outside your home? |  |  |  |
|  | Q: Did you wash your hands more frequently than usual? | 1 = yes  2 = no | 1 = 1 = yes  2 = 2 = no | SCSS20 |
|  | Q: Did you use special hand sanitizer or disinfection fluids more frequently than usual? |  |  |  |
|  | Q: Do you currently reduce your social contacts with people outside of your household because of Corona? | 1 = yes  2 = no | 1 = 1 = yes  2 = 2 = no | SCSAT20 |
|  | Q: How many times have you been tested for Covid-19? | 1 = not at all  2 = only once  3 = 2-5 times  4 = 6-10 times  5 = more than 10 times | 1 = = 1 not at all  2 = 2 = only once  3 = 3 = 2-5 times  4 = 4 = 6-10 times  5 = 5 = more than 10 times | SCSS21 |
| **reported health** | Q: Before the outbreak of Corona, would you say your health was …? | 1= excellent  2 = very good  3 = good  4 = fair  5 = poor | 1-2 = 1 excellent/very good  3 = 2= good  4-5 = 3= fair/poor | SCSS20 |
|  | Q: Would you say your health is …? |  |  | SCSS21 |
|  | GV: EURO- D (depression scale)  GV from Euro-D1 to Euro-D12 (sum score) | 1 = yes  0 = no | sum score: 0 (no depression) – 12 (major depression) | SCSAT20 |
|  | GV: GAD-7 (Generalized anxiety and depression scale)  GV from GAD 1a to GAD 1g (sum score) | 1 = not at all  2 = several days  3 = more than half the days  4 = nearly every day | sum score; for cut off points see Spitzer et al. (73):  0-4 = 1= minimal/ no anxiety  5-9 = 2 = low anxiety  10- 21 = 3 = moderate to severe anxiety | SCSAT20 |

**Continuation of table A.1**

| **Variable block** | **Question (Q) / Generated Variable (GV)** | **Manifestation in Survey** | **Recoded Manifestation in Analysis** | **Survey of origin** |
| --- | --- | --- | --- | --- |
| **health care utilization** | Q: Since the outbreak of Corona, did you forgo medical treatment because you were afraid to become infected by the corona virus? | 1 = yes  5 = no | 1 = 1= yes  5 = 2 = no | SCSS20 |
|  | Q: Since last interview/July 2020 did you forgo medical treatment because you were afraid to become infected by the corona virus? |  |  | SCSS21 |
|  | Q: Postponed medical appointment due to COVID-19? |  |  | SCSS20 |
|  | Q: Medical appointment postponed due to COVID-19 since last interview/July 2020? |  |  | SCSS21 |
|  | Q: Appointment for medical treatment denied since outbreak? |  |  | SCSS20 |
|  | Q: Appointment for medical treatment denied since last interview/July 2020? |  |  | SCSS21 |
|  | Q: Treated in hospital since last interview/July 2020? |  |  | SCSS21 |
|  | Q: Visited doctor/medical facility other than hospital since last interview/ July 2020? |  |  | SCSS21 |
| **social contact and support** | During the last three months, how often did you have personal contact, that is, face to face, with the following people from outside your home?  Q: Own Children? | 1 = daily  2 = several times a week  3 = about once a week  4 = less often  5 = never | 1 = 1 =daily  2 = 2 = several times a week  3 = 3 = about once a week  4 = 4 = less often  5 = 5 = never | SCSS21 |
|  | Q: Own grandchildren? |  |  |  |
|  | Q: Other non-relatives like neighbors/friends/colleagues? |  |  |  |
|  | During the last three months, how often did you have contact by phone, email or any other electronic means with the following people from outside your home?  Q: Own children? |  |  |  |
|  | Q: Other non-relatives like neighbors/friends/colleagues? |  |  |  |
|  | Since the outbreak of corona, were you helped by the following people from outside your home to obtain necessities, e.g. food, medications or emergency household repairs?  Q: Own Children? | 1 = yes  2 = no | 1 = 1 = yes  2 = 2 = no | SCSS21 |
|  | Q: Other relatives? |  |  |  |
|  | Q: Other non-relatives like neighbors/friends/colleagues? |  |  |  |

**Continuation of table A.1**

| **Variable block** | **Question / Generated Variable** | **Manifestation in Survey** | **Recoded Manifestation in Analysis** | **Survey of origin** |
| --- | --- | --- | --- | --- |
| **ICT use** | Q: Since the outbreak of Corona, have you used the Internet, for e-mailing, searching for information, making purchases, or for any other purpose at least once? | 1 = yes  5 = no | 1 = 1 = yes  5 = 2 = no | SCSS21 |
|  | If previous Q = yes  Nowadays, many things can be done online, that is, via the Internet. Since the outbreak of Corona, have you used the Internet more often, about the same, less often, or not at all for the following online activities?  Q: Searching for information on health-related issues? | 1 = more often  2 = about the same  3 = Less often  4 = not at all | 1 or 2 or 3 = 1 = yes  4 = 2 = no | SCSS21 |
|  | Q: Getting information about government services (for example for benefits, taxes, or passports) |  |  | SCSS21 |
|  | Q: Managing finances, such as online banking, paying bills, or paying taxes |  |  | SCSS21 |
|  | Q: Buying or selling goods or services online |  |  | SCSS21 |
|  | Q: Since the outbreak of Corona, how many remote medical consultations over the phone, computer, or any other electronic means, did you have, if any, with or without video? | numerical value (0 and above) | 1 to 10,00 = 1= yes at least once  0 = 2= no | SCSS21 |
| Example of reading: The first analysed variable or question in the questionnaire is: "How high do you consider your risk of catching Corona within the next 6 months?". Respondents could on a scale of 1 (very low) to 5 (very high). For analysis, data was recoded as follows: 1-2 into 1 = (very) low risk; 3 into 2 = medium risk; 4-5 into 3= (very) high risk, GV= generated variable, Q= question, SCSS20 = SHARE Corona Survey – summer 2020, SCSS21 = SHARE Corona Survey – summer 2021, SCSAT20= SHARE Corona Special Austria Survey- winter 2020 | | | | |

**Table A.2 - Health care utilization**

| **forwent medical treatment** | | | | | | |
| --- | --- | --- | --- | --- | --- | --- |
| **since outbreak (SCSS20)** | | | | **last interview/July 2020 (SSCS21)** | | |
|  | *non-poor* | *poor* | |  | *non-poor* | *poor* |
| *yes* | 13.7%_a_ | 13.4%_a_ | | *yes* | 8.3%_a_ | 7.4%_a_ |
| *no* | 86.3%_a_ | 86.6%_a_ | | *no* | 91.7%_a_ | 92.6%_a_ |
| *n* | 1525 | 337 | | *n* | 1523 | 336 |
| *Cramer's V* | 0.004 | | | *Cramer's V* | 0.013 | |
| *p* | 0.865 | | | *p* | 0.586 | |
| **postponed medical appointment due to COVID-19** | | | | | | |
| **since outbreak (SCSS20)** | | | | **last interview/July 2020 (SSCS21)** | | |
|  | *non-poor* | *poor* | |  | *non-poor* | *poor* |
| *yes* | 28.9%_a_ | 25.2%_a_ | | *yes* | 11.0%_a_ | 12.0%_a_ |
| *no* | 71.1%_a_ | 74.8%_a_ | | *no* | 89.0%_a_ | 88.0%_a_ |
| *n* | 1525 | 337 | | *n* | 1524 | 333 |
| *Cramer's V* | 0.032 | | | *Cramer's V* | 0.012 | |
| *p* | 0.173 | | | *p* | 0.604 | |
| **appointment for medical treatment denied** | | | | | | |
| **since outbreak (SCSS20)** | | | | **last interview/July 2020 (SSCS21)** | | |
|  | *non-poor* | *poor* | |  | *non-poor* | *poor* |
| *yes* | 4.7%_a_ | 3.9%_a_ | | *yes* | 2.4%_a_ | 2.1%_a_ |
| *no* | 95.3%_a_ | 96.1%_a_ | | *no* | 97.6%_a_ | 97.9%_a_ |
| *n* | 1525 | 336 | | *n* | 1525 | 335 |
| *Cramer's V* | 0.015 | | | *Cramer's V* | 0.009 | |
| *p* | 0.529 | | | *p* | 0.714 | |
| **treated in hospital since last interview/July 2020 (SCSS21)** | | | **visited doctor/medical facility other than hospital since last interview/ July 2020 (SCSS21)** | | | |
|  | *non-poor* | *poor* | |  | *non-poor* | *poor* |
| *yes* | 27.6%_a_ | 26.6%_a_ | | *yes* | 82.7%_a_ | 84.2%_a_ |
| *no* | 72.4%_a_ | 73.4%_a_ | | *no* | 17.3%_a_ | 15.8%_a_ |
| *n* | 1523 | 335 | | *n* | 1525 | 336 |
| *Cramer's V* | 0.009 | | | *Cramer's V* | 0.016 | |
| *p* | 0.69 | | | *p* | 0.497 | |
| The lower-case letters in the tables show the result of the *z*-test. aa = no significant difference between poor and non-poor; ab = significant difference between categories. We recommend interpreting the *z*-test only if the respective *chi²* test in the table is significant (when *p* <0,05). *Cramer’s V* measures the strength of the relationship between variables,  *n* =sample size, SCSS20 = SHARE Corona Survey – summer 2020, SCSS21 = SHARE Corona Survey – summer 2021 | | | | | | |

**Table A.3 – Social contact**

| **contact frequency with own children during last 3 months (SCSS21)** | | | | | **contact frequency with own grandchildren during last 3 months (SSCS21)** | | | | **contact frequency with neighbours/friends/colleagues during last 3 months (SSCS21)** | | | | | |
| --- | --- | --- | --- | --- | --- | --- | --- | --- | --- | --- | --- | --- | --- | --- |
|  | *non-poor* | | *poor* | |  | *non-poor* | | *poor* | |  | | *non-poor* | | *poor* |
| *daily* | 15.4%_a_ | | 19.3%_a_ | | *daily* | 7.7%_a_ | | 11.0%_a_ | | *daily* | | 4.1%_a_ | | 4.8%_a_ |
| *several times a week* | 26.2%_a_ | | 23.0%_a_ | | *several times a week* | 21.2%_a_ | | 18.3%_a_ | | *several times a week* | | 24.5%_a_ | | 25.1%_a_ |
| *about once a week* | 26.9%_a_ | | 28.2%_a_ | | *about once a week* | 23.7%_a_ | | 23.4%_a_ | | *about once a week* | | 30.3%_a_ | | 31.9%_a_ |
| *less often* | 27.0%_a_ | | 22.3%_a_ | | *less often* | 39.5%_a_ | | 35.9%_a_ | | *less often* | | 31.2%_a_ | | 25.1%_b_ |
| *never* | 4.5%_a_ | | 7.2%_a_ | | *never* | 7.9%_a_ | | 11.4%_a_ | | *never* | | 9.9%_a_ | | 13.1%_a_ |
| *n* | 1374 | | 305 | | *n* | 1159 | | 273 | | *n* | | 1521 | | 335 |
| *Cramer's V* | 0.075 | | | | *Cramer's V* | 0.074 | | | | *Cramer's V* | | 0.061 | | |
| *p* | 0.052 | | | | *p* | 0.101 | | | | *p* | | 0.146 | | |
| **electronic contact frequency with own children during last 3 months**  **(SCSS21)** | | | | | | | **electronic contact frequency with neighbours/friends/colleagues during last 3 months (SCSS21)** | | | | | | | |
|  | | *non-poor* | | *poor* | | |  | | | | *non-poor* | | *Poor* | |
| *daily* | | 20.3%_a_ | | 19.7%_a_ | | | *daily* | | | | 7.4%_a_ | | 7.8%_a_ | |
| *several times a week* | | 50.8%_a_ | | 48.5%_a_ | | | *several times a week* | | | | 39.9%_a_ | | 34.9%_a_ | |
| *about once a week* | | 16.4%_a_ | | 17.7%_a_ | | | *about once a week* | | | | 28.9%_a_ | | 27.8%_a_ | |
| *less often* | | 7.4%_a_ | | 8.2%_a_ | | | *less often* | | | | 19.3%_a_ | | 22.7%_a_ | |
| *never* | | 5.2%_a_ | | 5.9%_a_ | | | *never* | | | | 4.5%_a_ | | 6.9%_a_ | |
| *n* | | 1373 | | 305 | | | *n* | | | | 1518 | | 335 | |
| *Cramer's V* | | 0.025 | | | | | *Cramer's V* | | | | 0.6 | | | |
| *p* | | 0.903 | | | | | *p* | | | | 0.155 | | | |
| The lower-case letters in the tables show the result of the *z*-test. aa = no significant difference between poor and non-poor; ab = significant difference between categories. We recommend interpreting the *z*-test only if the respective *chi²* test in the table is significant (when *p* <0,05). *Cramer’s V* measures the strength of the relationship between variables,  *n* =sample size, SCSS20 = SHARE Corona Survey – summer 2020, SCSS21 = SHARE Corona Survey – summer 2021 | | | | | | | | | | | | | | |
